# Supplementary material for: Accurate inference of genome-wide spatial expression with iSpatial
Source: Sci Adv. 2022 Aug 26;8(34):eabq0990. doi: 10.1126/sciadv.abq0990 (PMC9417177; doi:10.1126/sciadv.abq0990)
Supplement: Supplementary file 1 — Figs. S1 to S11 [file sciadv.abq0990_sm.pdf]

Science Advances
AAAS

Supplementary Materials for

Accurate inference of genome-wide spatial expression with iSpatial

Chao Zhang *et al*.

Corresponding author: Yi Zhang, yzhang@genetics.med.harvard.edu

Sci. Adv. **8**, eabq0990 (2022)
DOI: 10.1126/sciadv.abq0990

This PDF file includes:

Figs. S1 to S11

Supplementary Figures

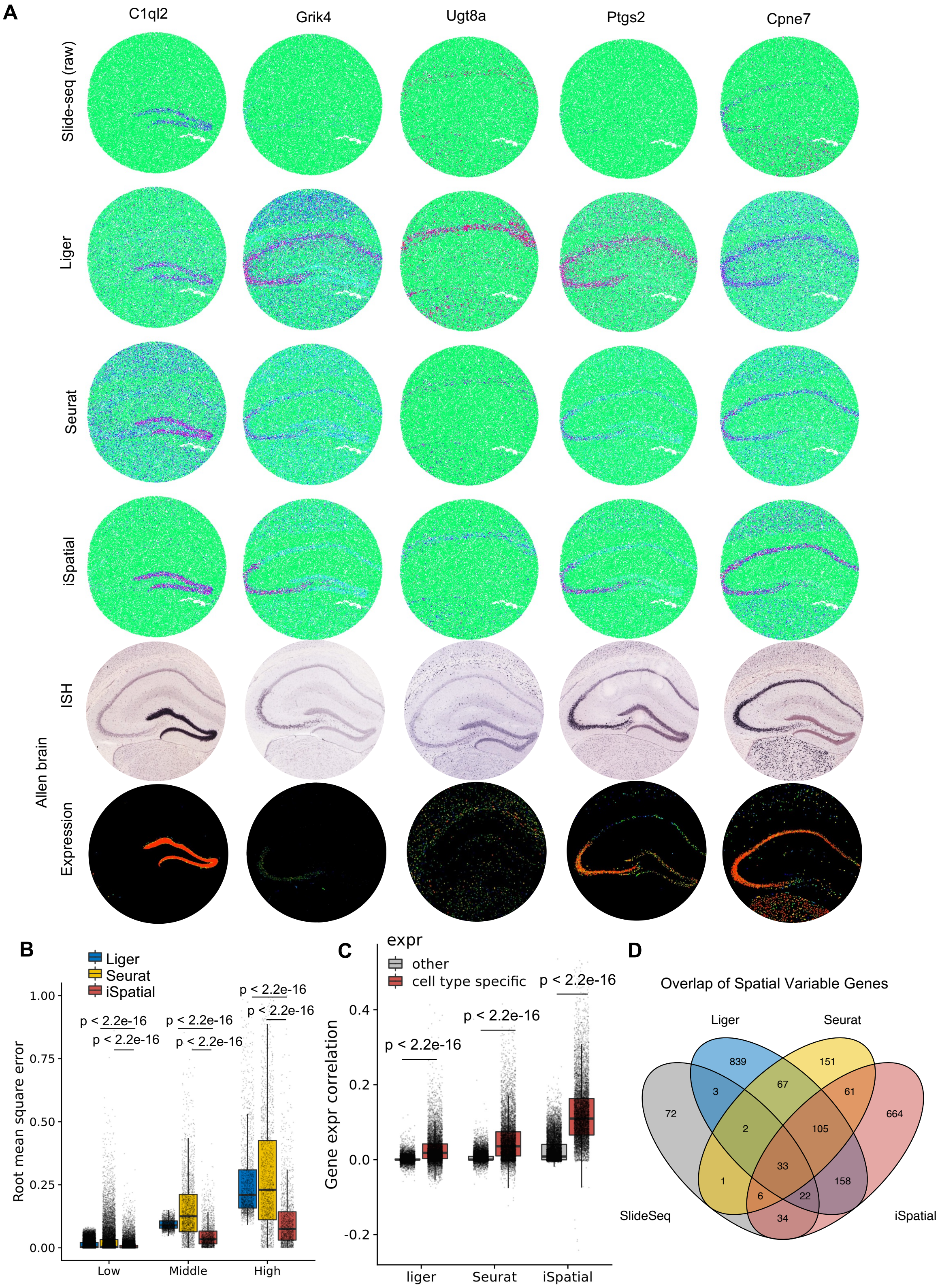

fig. S1: Benchmarking the performance of iSpatial in inferring genome-wide spatial transriptome. **A**, Representative examples showing the performance of Liger, Seurat and iSpatial in inferring spatial transcriptome. **B**, The RMSE between raw expression value and inferred value of Slide-seq V2 data. **C**, The gene expression correlation between raw data and Liger, Seurat, or iSpatial inferred data of cell type specific genes or other genes. **D**, The overlaps of the spatial variable genes detected by different methods.

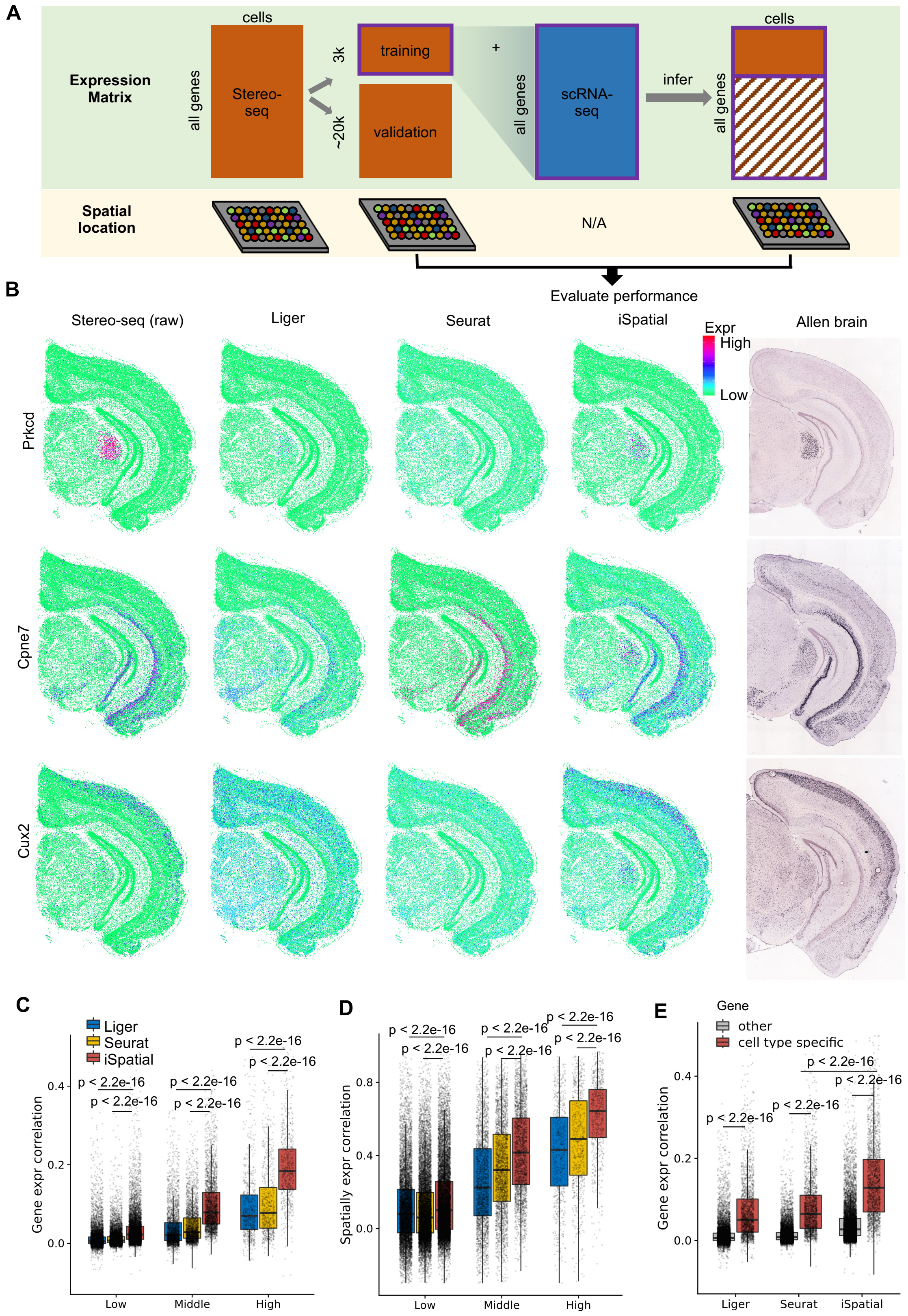

A
Expression Matrix
cells
all genes
Stereo-seq
3k
~20k
training
validation
+
all genes
scRNA-seq
infer
cells
all genes
Spatial location
N/A
Evaluate performance
B
Stereo-seq (raw)
Liger
Seurat
iSpatial
Expr
High
Low
Allen brain
Prkcd
Cpne7
Cux2
C
Liger
Seurat
iSpatial
p < 2.2e-16
Gene expr correlation
Low
Middle
High
D
Spatially expr correlation
E
Gene
other
cell type specific
Liger
Seurat
iSpatial

fig. S2: Benchmarking the performance on Stereo-seq data. A, A graphical illustration of the evaluation procedure. **B**, Representative examples showing the raw or inferred spatial expression by Liger, Seurat and iSpatial. Allen brain ISH data are also shown. **C**, The gene expression correlation between Stereo-seq raw data and Liger, Seurat, or iSpatial inferred data. The validation genes are divided into 3 groups based on their expression levels. **D**, The spatial expression correlation between inferred and raw Stereo-seq data. **E**, The gene expression correlation between Stereo-seq raw data and Liger, Seurat, or iSpatial inferred data of cell type specific genes or other genes. Two-side Wilcoxon Rank Sum test was used.

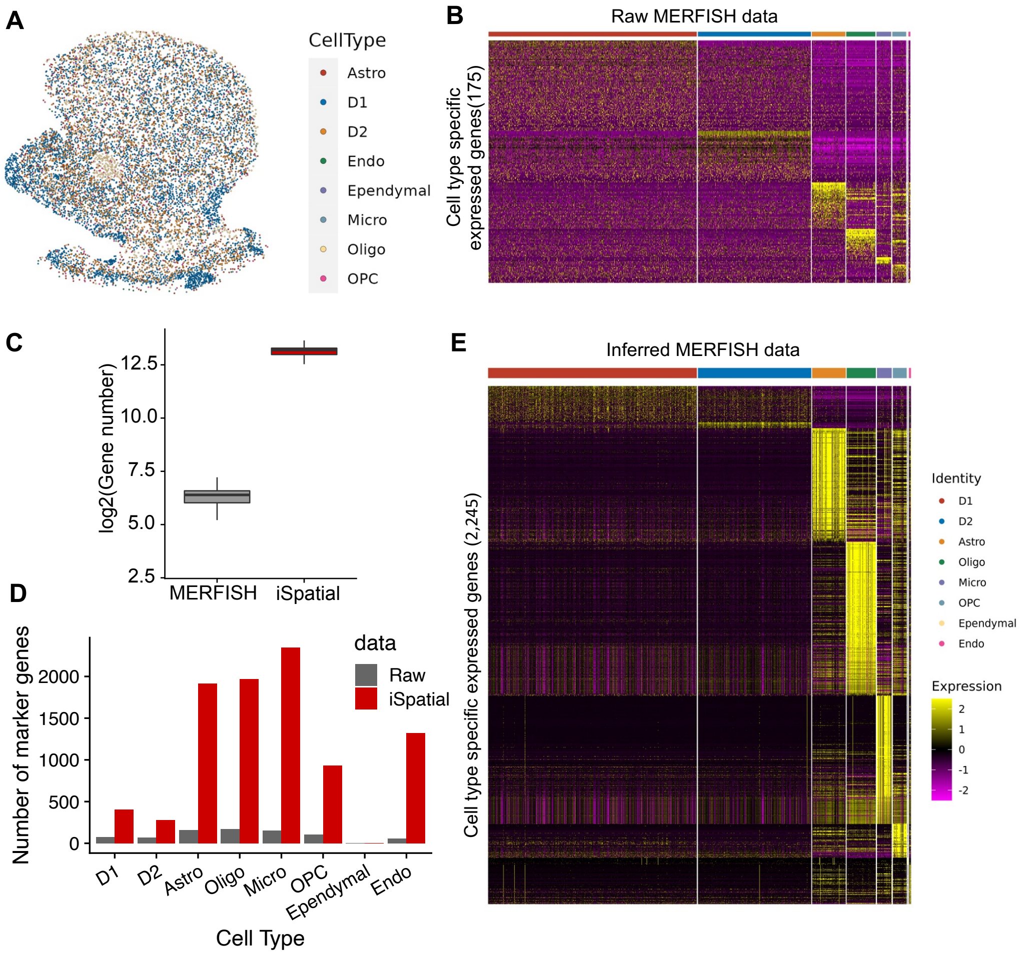

fig. S3: iSpatial is applicable to MERFISH dataset. **A**, Spatial distribution of the cell types detected by MERFISH. **B**, The heatmap of the 175 cell type markers detected in the raw MERFISH data. **C**, The numbers of detected genes in raw MERFISH data and after interfering by iSpatial. **D**, The number of cell type specific expressed genes in each cell type detected in raw and iSpatial inferred data. **E**, The heatmap of all maker genes detected after inferring by iSpatial.

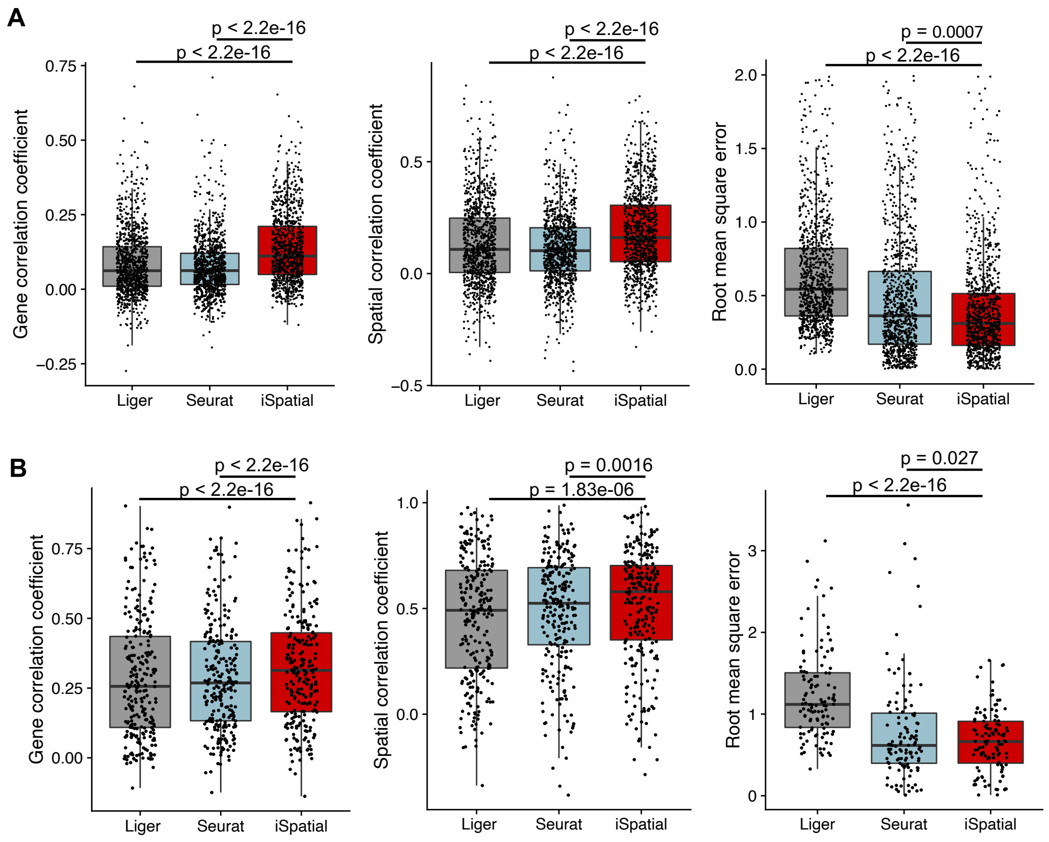

fig. S4: Ten-fold cross validation on the performance of the three methods in mouse cortex (A) and striatum (B). Left panels: gene expression correlation between raw expression data and inferred data. Middle panels: spatial expression correlation between raw expression data and inferred data. Right panels: The RMSE between raw expression data and inferred data.

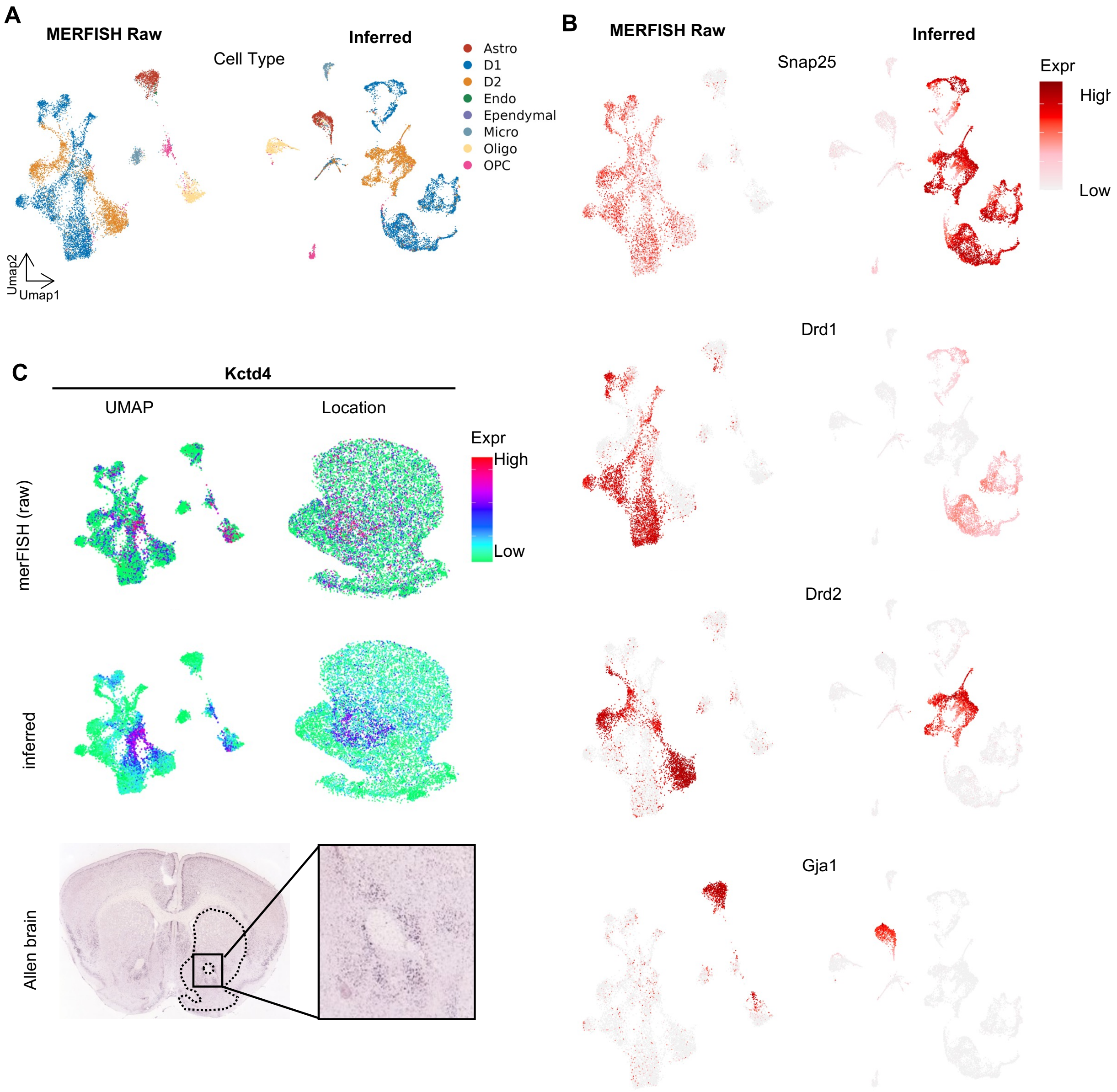

fig. S5: iSpatial can reduce false-positive and false-negative signals in the original MERFISH data. A, Cell type clusters on the UMAPs derived from raw or inferred data. **B**, UMAPs showing the expression of representative cell type marker genes. The UMAPs are generated from raw or iSpatial inferred expression profiles, respectively. Marker genes for neuron (*Snap25*), D1 medium spiny neuron (*Drd1*), D2 medium spiny neuron (*Drd2*), and astrocytes (*Gja1*) are presented. **C**, The UMAP and spatial expression of *Kctd4* in the raw MERFISH (top panels) and inferred by iSpatial (middle panels) compared with the ISH data from the ABA (bottom panel).

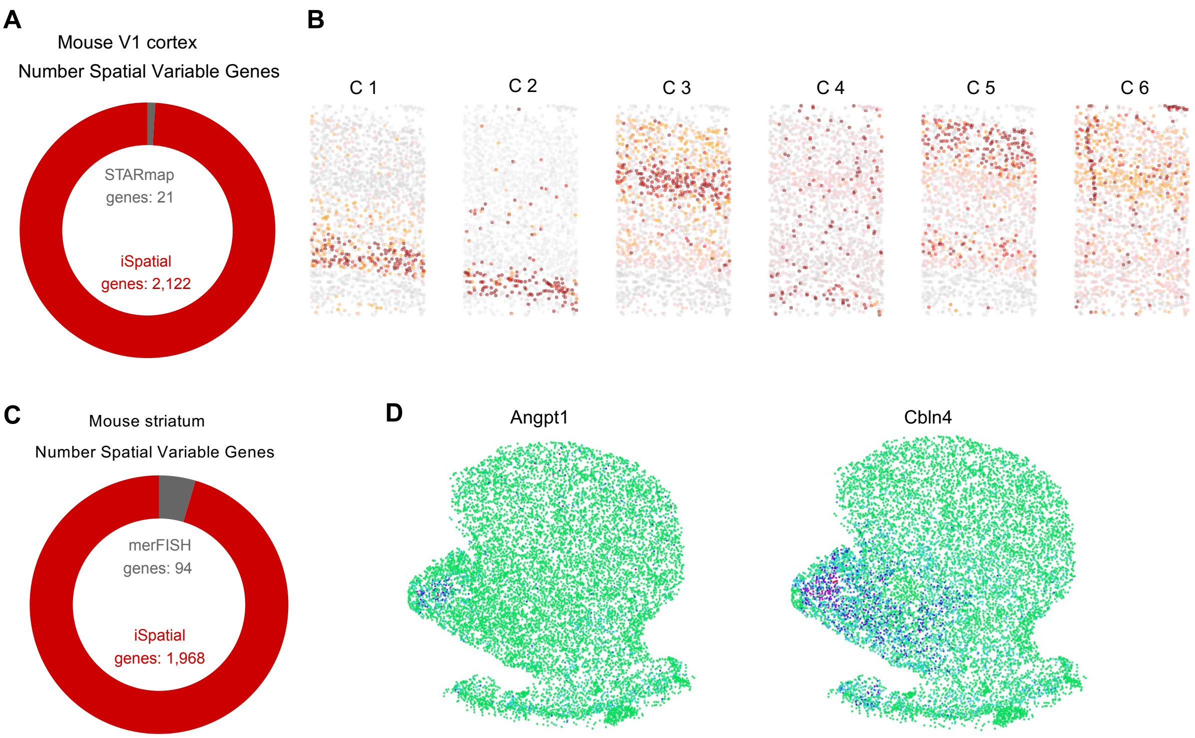

fig. S6: iSpatial detects spatial variable genes in mouse cortex (A, B) and striatum (C, D). **A**, The number of spatial variable genes in mouse V1 cortex detected by STARmap and after iSpatial interferring. **B**, The layer distribution of the 6 clusters spatial variable genes. The plot is colored by the average expression level of the genes in each cluster. **C**, The number of spatial variable genes in mouse striatum detected by MERFISH and after iSpatial interfering. **D**, Inferred spatial expression signals of representative genes in the C8 cluster.

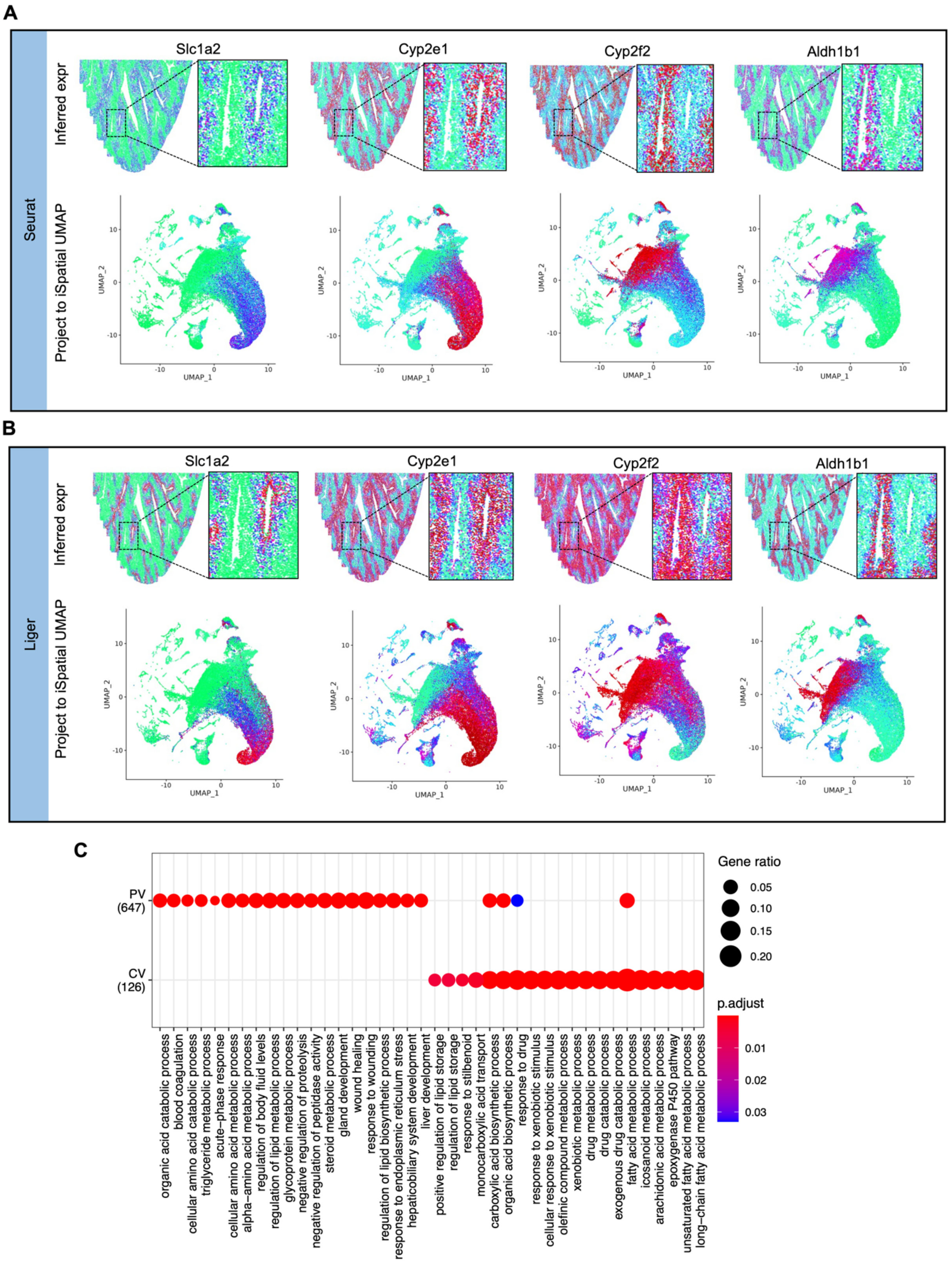
A
Seurat
Inferred expr
Project to iSpatial UMAP
Slc1a2
Cyp2e1
Cyp2f2
Aldh1b1
UMAP_1
UMAP_2
B
Liger
Inferred expr
Project to iSpatial UMAP
Slc1a2
Cyp2e1
Cyp2f2
Aldh1b1
UMAP_1
UMAP_2
C
PV (647)
CV (126)
Gene ratio
0.05
0.10
0.15
0.20
p.adjust
0.01
0.02
0.03
organic acid catabolic process
blood coagulation
cellular amino acid catabolic process
triglyceride metabolic process
acute−phase response
cellular amino acid metabolic process
alpha−amino acid metabolic process
regulation of body fluid levels
regulation of lipid metabolic process
glycoprotein metabolic process
negative regulation of proteolysis
negative regulation of peptidase activity
steroid metabolic process
gland development
wound healing
response to wounding
regulation of lipid biosynthetic process
response to endoplasmic reticulum stress
hepaticobiliary system development
liver development
positive regulation of lipid storage
regulation of lipid storage
response to stilbenoid
monocarboxylic acid transport
carboxylic acid biosynthetic process
organic acid biosynthetic process
response to drug
response to xenobiotic stimulus
cellular response to xenobiotic stimulus
olefinic compound metabolic process
xenobiotic metabolic process
drug metabolic process
drug catabolic process
exogenous drug catabolic process
fatty acid metabolic process
icosanoid metabolic process
arachidonic acid metabolic process
epoxygenase P450 pathway
unsaturated fatty acid metabolic process
long−chain fatty acid metabolic process

fig. S7: iSpatial infers the spatial expression patterns in liver. **A, B**, Examples of spatial expressed genes enriched in CV (*Slc1a2*, *Cyp2e1*) or PV (*Cyp2f2*, *Aldh1b1*), inferred by Seurat (A) and Liger (B). The upper panels show the spatial expression patterns. The bottom panels show the inferred genes' expression by the indicated method projects on the iSpatial UMAP. **C**, The top 20 enriched Gene Ontology terms of genes selectively expressed near CV or PV.

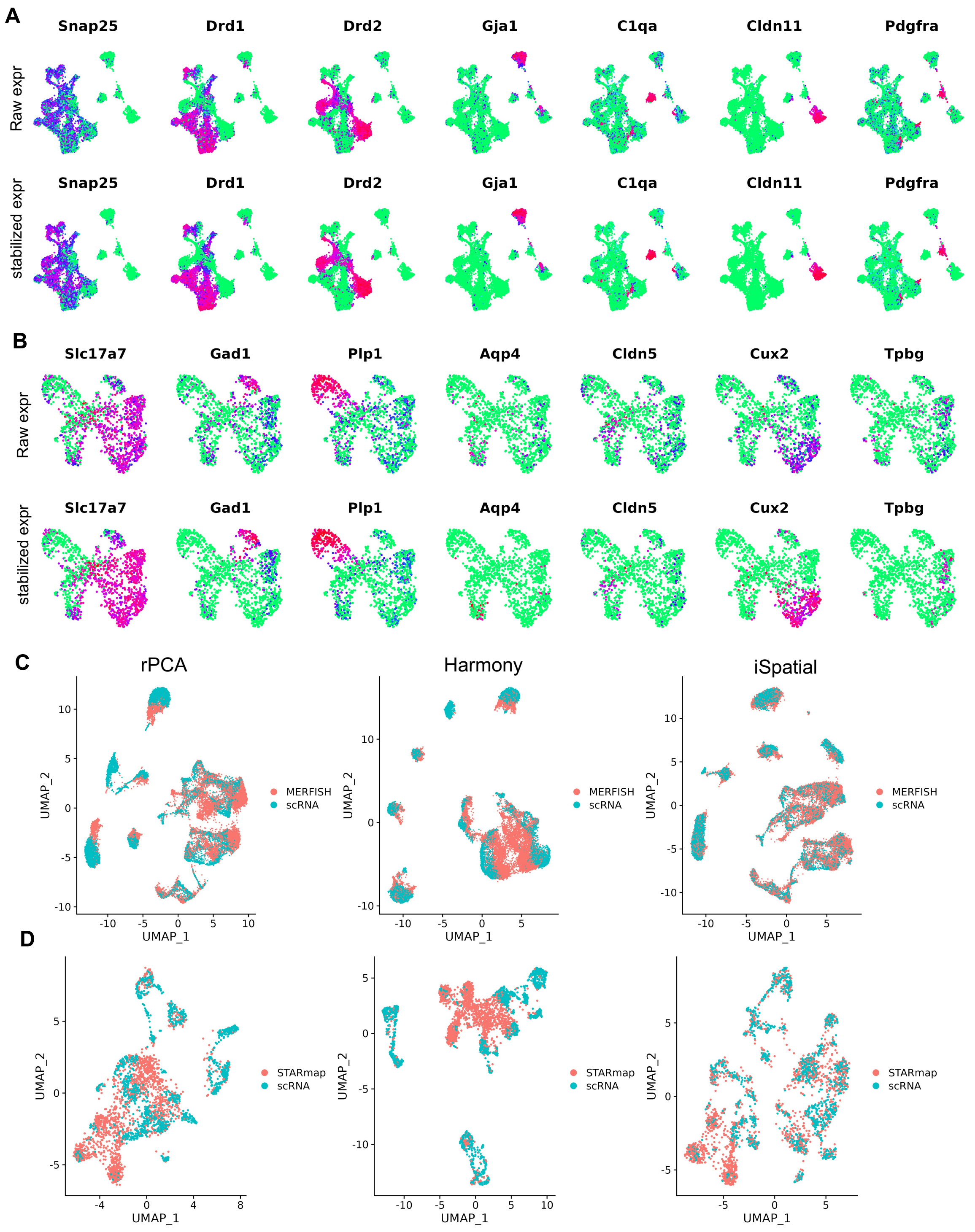

fig. S8: Stabilization and two-rounds integration steps contribute to the improved performance of iSpatial. A, B, UMAPs showing the expression signals of representative markers before (top panels) and after (bottom panels) expression stabilization in mouse striatum

(**A**) or cortex (**B**) data. **C, D**, The co-embed UMAPs showing the integration of ST and scRNA-seq data in mouse striatum (**C**) or cortex (**D**) data.

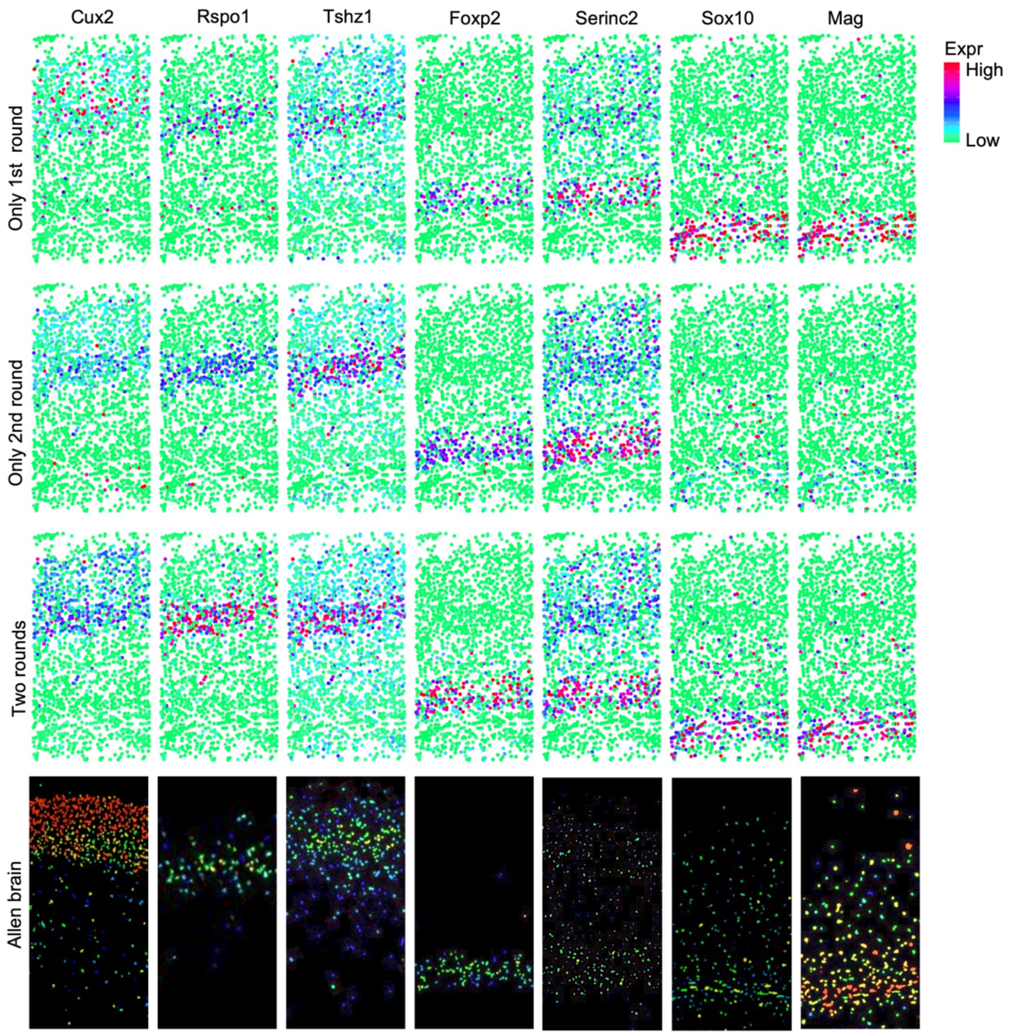

fig S9: Comparing the two-rounds integration with one round. The spatial expression of the representative genes of the mouse cortex raw STARmap data inferred by one round (top two rows) or two rounds (third row) of integration by iSpatial compared with the ISH data from the ABA (bottom row). Reciprocal PCA is used for the first-round integration, and Harmony is used for the second-round integration.

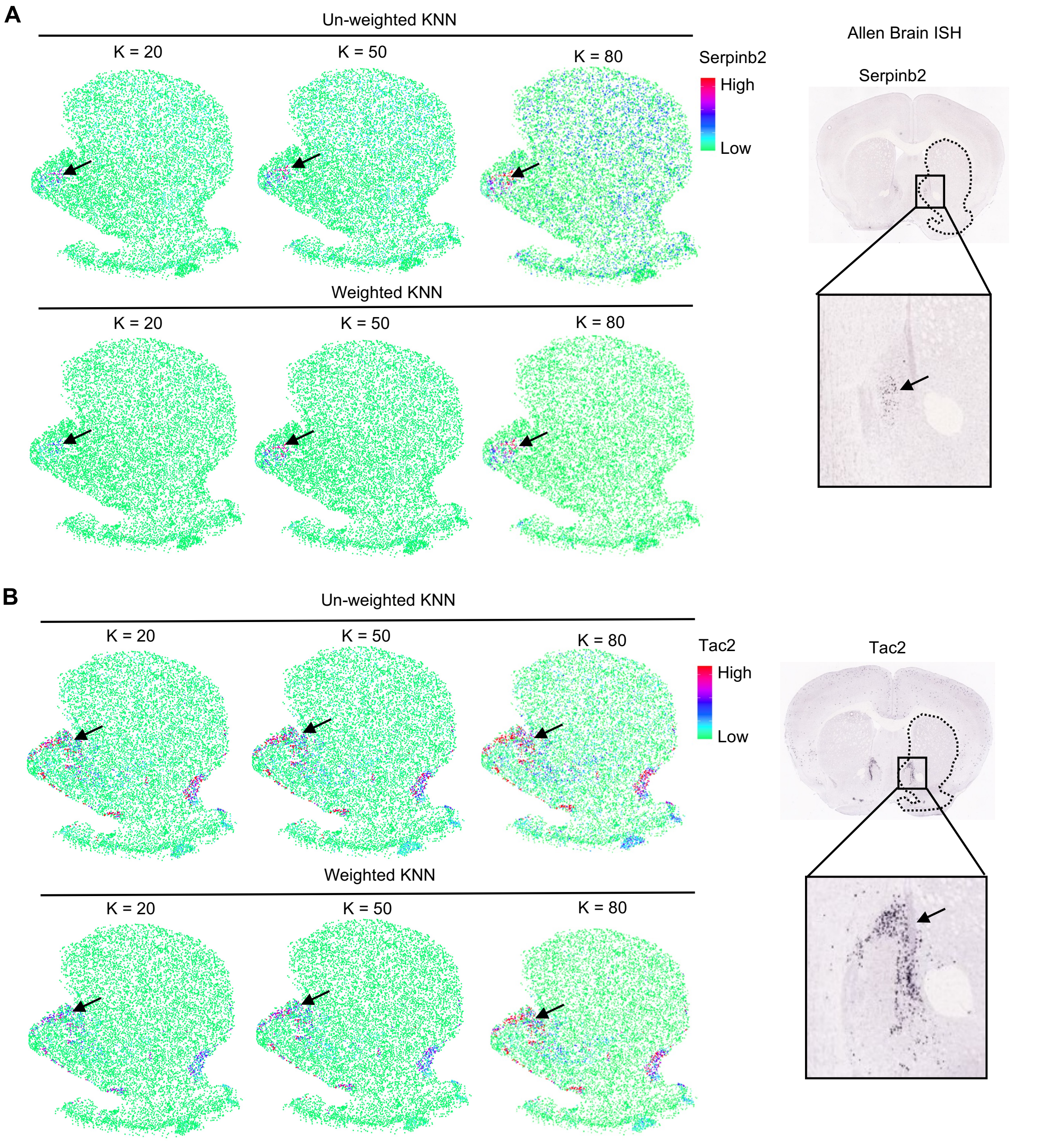

fig S10: Comparing the performance of weighted or un-weighted KNN. A, B, The inferred spatial expression of *Serpinb2* (**A**) and *Tac2* (**B**) in rare cell population using un-weighted KNN or weighted KNN under different K value. Allen brain ISH data were showed on right. Arrows indicate the location of the rare cell population.

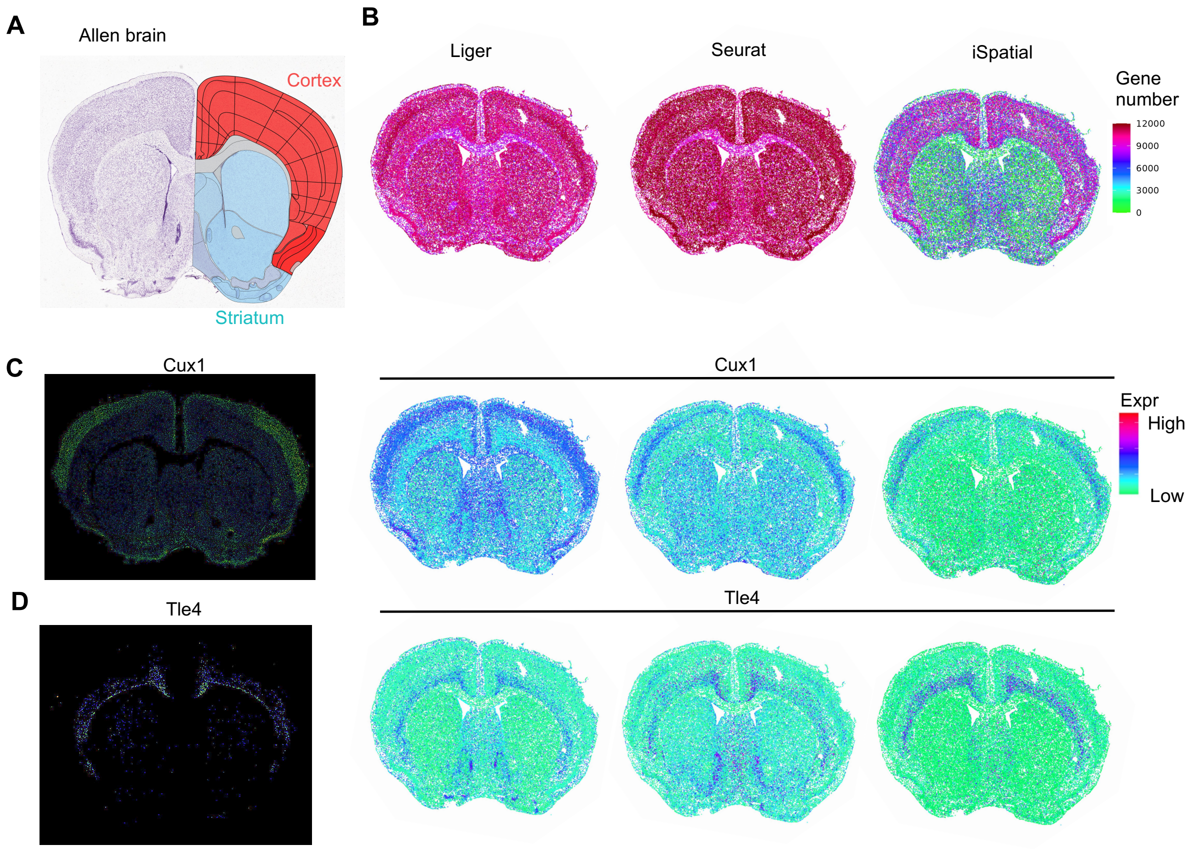

fig. S11: Infer spatial expression by unmatched ST and scRNAseq data. A, Schematic of the anatomic region of mouse cortex and striatum. **B**, Varied number of measured genes in each cell of a whole brain slice inferred by Liger, Seurat and iSpatial. Unmatched MERFISH and scRNA-seq data are used. MERFISH data covers the entire slice. scRNA-seq data only covers cortex region. **C and D**, Representative examples showing the performance of Liger, Seurat and iSpatial in inferring spatial transcriptome with unmatched scRNA-seq data. Allen brain ISH data were showed in left.
